# Bayesian Multi-Trait Analysis Reveals a Useful Tool to Increase Oil Concentration and to Decrease Toxicity in *Jatropha curcas* L.

**DOI:** 10.1371/journal.pone.0157038

**Published:** 2016-06-09

**Authors:** Vinícius Silva Junqueira, Leonardo de Azevedo Peixoto, Bruno Galvêas Laviola, Leonardo Lopes Bhering, Simone Mendonça, Tania da Silveira Agostini Costa, Rosemar Antoniassi

**Affiliations:** 1 Animal Science Department, Federal University of Viçosa, Viçosa, Minas Gerais, zipcode: 36570–000, Brazil; 2 Biology Department, Federal University Viçosa, Viçosa, Minas Gerais, zipcode: 36570–000, Brazil; 3 Empresa Brasileira de Pesquisa Agropecuária, Embrapa Agroenergia, Parque Estação Biológica–PqEB s/n, Asa Norte, zipcode: 70770901, Brasília, Brazil; 4 Empresa Brasileira de Pesquisa Agropecuária, Centro Nacional de Pesquisa de Recursos Genéticos e Biotecnologia, Parque Estação Biológica, PqEB, W5 Norte, Final PqEB, zipcode: 70770917, Brasília, Brazil; 5 Empresa Brasileira de Pesquisa Agropecuária, Centro Nacional de Pesquisa de Tecnologia Agroindustrial de Alimentos, Avenida das Americas 29501 Guaratiba, zipcode: 23020470, Rio de Janeiro, Brazil; College of Agricultural Sciences, UNITED STATES

## Abstract

The biggest challenge for jatropha breeding is to identify superior genotypes that present high seed yield and seed oil content with reduced toxicity levels. Therefore, the objective of this study was to estimate genetic parameters for three important traits (weight of 100 seed, oil seed content, and phorbol ester concentration), and to select superior genotypes to be used as progenitors in jatropha breeding. Additionally, the genotypic values and the genetic parameters estimated under the Bayesian multi-trait approach were used to evaluate different selection indices scenarios of 179 half-sib families. Three different scenarios and economic weights were considered. It was possible to simultaneously reduce toxicity and increase seed oil content and weight of 100 seed by using index selection based on genotypic value estimated by the Bayesian multi-trait approach. Indeed, we identified two families that present these characteristics by evaluating genetic diversity using the Ward clustering method, which suggested nine homogenous clusters. Future researches must integrate the Bayesian multi-trait methods with realized relationship matrix, aiming to build accurate selection indices models.

## Introduction

Jatropha (*Jatropha curcas* L.) has many economically interesting characteristics, and nowadays, it has been considered as the most important shrub for biodiesel production, mainly due to the large amount of oil content it produces [1]. Additionally, jatropha stands out due to premature production period, when it is compared with other palms commonly used for biofuel production [2]. Moreover, this culture presents drought resistance [3], low seed cost [4], high seed oil content [5], and easy adaptation [2].

Approximately 35% of seeds’ content is composed of oil, of which 24.6% is crude protein and 47.2% is crude fat [6]. Moreover, jatropha’s oil presents higher oxidation stability than soybean’s oil; lower viscosity than castor’s oil; and lower pour point than other palms [7].

Despite the large amount of oil and crude protein content, consumption of seeds can represent a risk for animal health [8]. Indeed, the use of jatropha’s cake (by-product of seeds industrial processing) as animal feed, and consequently the crop cultivation economic viability are conditioned by the low toxicity content [9]. Phorbol ester has been considered as the main substance for jatropha’s seeds toxicity [10], and has been differently reported in toxic genotypes (2 to 6 mg/g of dry matter) and in non toxic genotypes (0 to 1.8 mg/g) [9]. Thus, there is the need to achieve highly productive genotypes with respect to high seed oil content and low level of toxicity. Therefore, the use of breeding techniques must be adopted in order to identify superior genotypes aiming at the improvement for these traits.

Bayesian multi-trait models have become useful statistics method for plant and animal genetic evaluations. Many authors have shown that this model is more flexible and effective than the least squares method, since it is not only based on the likelihood function, but it also allows *a priori* knowledge assumption when defining prior distribution [11].

Many previous studies have estimated variance components and genetic parameters under different statistical methods in jatropha [12–15]. However, none of them carried out multi-trait analysis using a Bayesian approach for seed oil content (**SOC**, %), weight of 100 seeds (**W100S**, g), and phorbol ester concentration (**PEC**, mg/g). Therefore, the Bayesian multi-trait analysis was carried out in order to estimate variance components and genetic parameters, which were used to evaluate genetic diversity and selection indices, aiming to identify superior genotypes for **SOC**, **W100S** and **PEC** traits.

## Materials and Methods

### Experimental design

The experiment considered the evaluation of 179 jatropha half-sib families from the Embrapa Cerrados germplasm bank [samples were collected in different Brazilian regions (S1 Table)]. It is settled in the experimental field of Embrapa Cerrados, Planaltina, Distrito Federal, Brazil (15°35’30”S and 47°42’30”W; 1007 m asl). The experiment was implemented in November, 2008, in a complete randomized block design with 2 replications, and 5 plants per plot, arranged in rows, spaced 4 m between rows, and 2 m between plants. All management practices were based on Dias et al. [16], and they were adapted according to recent research advances regarding jatropha in Brazil [17–19]. The half-sib families were evaluated over 5 crop years (2010 to 2014) for **W100S,** while **SOC** and **PEC** were evaluated only in 2014. All data used in this study are available in Table in S2 Table.

Phorbol ester was extracted according to procedure described by Makkar et al. [20]. Milled seeds was carried on in accelerated solvent extraction equipment called Dionex (model ASE 350). Tetrahydrofuran was used as solvent, and posteriorly, it was evaporated under nitrogen flow. The Oily residual was transferred to a test tube (10 mL), and extracted repeatedly (four times) using methanol (once using 2mL and three using 1mL). Finally, the oily residual was transferred to volumetric flask (5 mL). The work solution was filtered using VertiPure PTFE Syringe (13 mm, 0.2 μm) and 1oo mL of this solution was injected into High Performance Liquid Chromatography (HPLC). A typical column (C18 250 x 4.6 mm) was used, with the temperature around 25°C. A UV detector allowed on-column detection operated from 200 nm to 340 nm. The standard curve was built using 12-myristate 13—phorbol Acetate.

The oil extraction was performed crushing seeds and albumens, and weighing epicarp and mesocarp separately. Analyses were performed according to Adolfo Lutz Institute protocols. Sample (200 g of seeds) for each plant and replicated twice. Soxhlet was used to extract the oil and the hexane was used as solvent.

Despite the five consecutive years of evaluations of **W100S**, only the records of 2014 were used because **SOC** and **PEC** traits were recorded only in this specific year. Additionally, mean phenotypes were used for **W100S**, and these records were adjusted according to the number of replications within genotypes and blocks. Also, all analyses were carried out using 358 phenotypic records for each trait of the 179 half-sib families.

### Statistical model and analysis

Variance components and genetic parameter estimates were obtained under the Bayesian approach, via Gibbs sampling, using the Gibbs2f90 software, as described by Misztal et al. [21]. We considered a total of 100,000 cycles after discarding the 40,000 initial samples used for burn-in and thinned every tenth iteration, resulting in a total of 6,000 samples. The convergence of Markov Chain Monte Carlo (**MCMC**) was tested by the Geweke criterion [22], using two packages: boa [23], and CODA [24], implemented in the R software [25]. Posterior means, key percentiles and standard deviations (**SD**) for estimated parameters were obtained from MCMC samples. Multi-trait mixed model was:

$$y_{ijkl}=\mu_{i}+b_{ik}+g_{ij}+e_{ijkl}$$ [1]

where *y*_*ijkl*_ is the *l*^*th*^ = {1,2,…,358} phenotypic value of *i*^*th*^ = {1,2,3} trait, on *j*^*th*^ = {1,2,…,179} genotype, within *k*^*th*^ = {1,2} blocks; *μ*_*i*_ is the overall mean of *i*^*th*^ trait; and *e*_*ijkl*_ is the residual term.

Under the Bayesian approach, the following joint distribution of data (likelihood function) were:

$$y_{ij}|\beta ,g,G_{0},R_{0}∼N(x\prime_{ij}\beta +z\prime_{ij}g,\sigma_{e}^{2})$$ [2]

where **β** is the vector of *a prior* probability of systematic effects (overall mean and blocks for each trait from Eq [1]); **g** = {*g*_*ij*_}∼*N*(**0**,**I**⨂**G**_0_) is the vector of *a prior* probability of genotypic values, where **I** is the identity matrix and $G_{0}=\left[\begin{array}{l} \sigma_{a_{1}}^{2}\;\sigma_{a_{12}}\;\sigma_{a_{13}}\\ \sigma_{a_{21}}\;\sigma_{a_{2}}^{2}\;\sigma_{a_{23}}\\ \sigma_{a_{31}}\;\sigma_{a_{32}}\;\sigma_{a_{3}}^{2} \end{array}\right]$ is the genotypic variance matrix; **e** = {*e*_*ijkl*_}∼*N*(**0**,**I**⨂**R**_0_) is the vector of *a prior* probability of residual values with normal independent identical distribution; where $R_{0}=\left[\begin{array}{l} \sigma_{e_{1}}^{2}\;\sigma_{e_{12}}\;\sigma_{e_{13}}\\ \sigma_{e_{21}}\;\sigma_{e_{2}}^{2}\;\sigma_{e_{23}}\\ \sigma_{e_{31}}\;\sigma_{e_{32}}\;\sigma_{e_{3}}^{2} \end{array}\right]$; **x**′_*ij*_ and **z**′_*ij*_ are incidence rows that relates systematic and genotypes effects within traits to the respective phenotypic value; and $\sigma_{e}^{2}$ is residual variance assumed as homogeneous.

The following *a priori* probability distributions for the location parameters of interest were given by:

$$\beta \sim N(b_{0},V_{b})$$ [3]

where **V**_*b*_ is a diagonal matrix of the *a priori* variance of **β**, assuming **V**_*b*_ → ∞;

Three-dimensional scaled inverted Wishart distributions are assigned as prior process for each of the **G**_0_ and **R**_0_ covariance matrices:

$$G_{0}\sim W_{3}^{-1}(\Sigma_{g},n)$$ [4]

$$R_{0}\sim W_{3}^{-1}(\Sigma_{e},n)$$ [5]

Where **Σ**_g_ and **Σ**_e_ are scale matrices, and *n* = 5 is the degree of freedom parameter.

The joint posterior density of all parameters, which are dependent on the genotypic effects of the corresponding matrix, but which take over prior independence, is given by

$$p(\beta ,g,G_{0},R_{0}|y)\propto p(y|\beta ,g,G_{0},R_{0})p(\beta |b_{0},V_{b})p(g|I⨂G_{0})p(G_{0}|\Sigma_{g},n)p(R_{0}|\Sigma_{e},n)$$ [6]

### Clustering and genetic diversity

A cluster analysis was carried out aiming to understand the genotypic relationship between traits, and to identify genotypes that expressed genetically similar performance. Hence, we intended to cluster homogeneous genotypes. The first step of cluster analysis is to calculate dissimilarity (**D**) matrix. In this study, we adopted the Mahalanobis distance, which can be defined as follows:

$$d^{2}(g_{1},g_{2})={\left(g_{1}-g_{2}\right)}^{T}\Psi (g_{1}-g_{2})$$ [7]

Where *g*_1_ and *g*_2_ are genotypic values for **SOC**, **W100S** or **PEC** traits; Ψ = **S**^−1^ is the Mahalanobis generalized distance; and **S**^−1^ is the three-dimensional inverse of variance matrix. This metric was used to consider the covariance between genotypes evaluated in different blocks.

Thus, the Ward cluster hierarchy method was applied, and it was chosen aiming to maximize the homogeneity within clusters so that the sum of square of error (**SSE**) is minimum. SSE of each cluster can be achieved as follows:

$$SSE_{c_{s}=}\sum_{s=1}^{n_{m}}{\left(g_{s}^{\left(m\right)}-{\bar{g}}_{.}^{\left(m\right)}\right)}^{T}(g_{s}^{\left(m\right)}-{\bar{g}}_{.}^{\left(m\right)})$$ [8]

where *n*_*m*_ is the number of individuals on *m*^*th*^ cluster; and $n=\sum_{m=1}^{t}n_{m}$

Mojena criteria was used to set the optimal number of clusters, and the method is based on computing the highest amplitude between clusters that maximizes the quality of the clustering [26].

$$\alpha_{j}>\bar{\alpha }+\omega S_{\alpha }$$ [9]

where *j* = (1,2,…,*n*) is the number of clusters; *α*_*j*_ is the correspondence joint point to *n* − *j* + 1 clusters; $\bar{\alpha }$ and *S*_*α*_ are the mean and the standard deviation of *α*′s; and *ω* is a constant equal to 1.25, as suggest by [27].

### Selection index calculations

Selection indices procedures were constructed according to Hazel [28]. Overall genetic gain can be achieved by selecting individuals by the sum of its several genotypes for each trait weighted by its relative economic value. This aggregate genotype (**H**) can by defined as:

$$H=g_{1}w_{1}+g_{2}w_{2}+\cdots +g_{n}w_{n}$$ [10]

Where **g**_*i*_, *i* = {1,2,…,*n*} is the vector of genotypic values for the *n*^*th*^ trait; and w_*i*_, *i* = {1,2,…,*n*} is the relative economic value.

In this study, three different scenarios were used, and they included different traits in the selection indices. Additionally, three different weights (**w**) of the traits in the breeding goal were taken into account. In the first situation, all traits had weight of 1 monetary unit per genetic standard deviation. In the second situation, **SOC** received weight of 2 and 1 monetary units for the other traits. Finally, we set weight of 4 monetary units for **SOC**, 2 for **W100S,** and 1 for **PEC,** in the third scenario. Traits contemplated in these scenarios were defined to depict a situation in which an established breeding program already working on selection for **SOC** intends to incorporate **W100S** and **PEC** in its breeding goal. As a trait of major importance, only **SOC** was used as target in the first scenario. The next two scenarios gradually included **W100S** and **PEC** in selection indices.

The set up matrices **P**, **G** and **C** contains phenotypic (co)variance between all components in a given scenario; covariance between traits of selection index and additive genetic values for traits of the breeding goal; and genetic (co)variance between traits in the breeding goal, respectively. Selection indices coefficients were calculated by **b** = **P**^**−1**^**Gw**, where **w** is the vector of relative economic weights expressed in monetary units per measurement units of the traits. Variances of the index (**I**) and of the H were calculated by $\sigma_{I}^{2}=b\prime Pb$ and $\sigma_{H}^{2}=w\prime Cw$. Correlation between the index and the genotypic aggregate (accuracy of the index) was calculated by $R_{IH}=\frac{\sigma_{I}}{\sigma_{H}}$.

Monetary overall genetic gain per generation was calculated by Δ*G* = (*i*)*R*_*IH*_*σ*_*H*_, and the response to selection per generation (**S**) for each trait was calculated by:

$$S=\frac{i}{\sigma_{I}}b\prime G$$ [11]

where *i* is the selection intensity assumed to be 1.75, considering the selection of the 10% superior genotypes.

## Results

In this study, we evaluated 179 genotypes for the three important traits in jatropha breeding program: seed oil content (**SOC**), weight of 100 seeds (**W100S**) and phorbol ester concentration (**PEC**) aiming to identify superior half-sib families under a Bayesian multi-trait model, using selection indices procedures.

### Phenotypic evaluation

**Fig 1:**
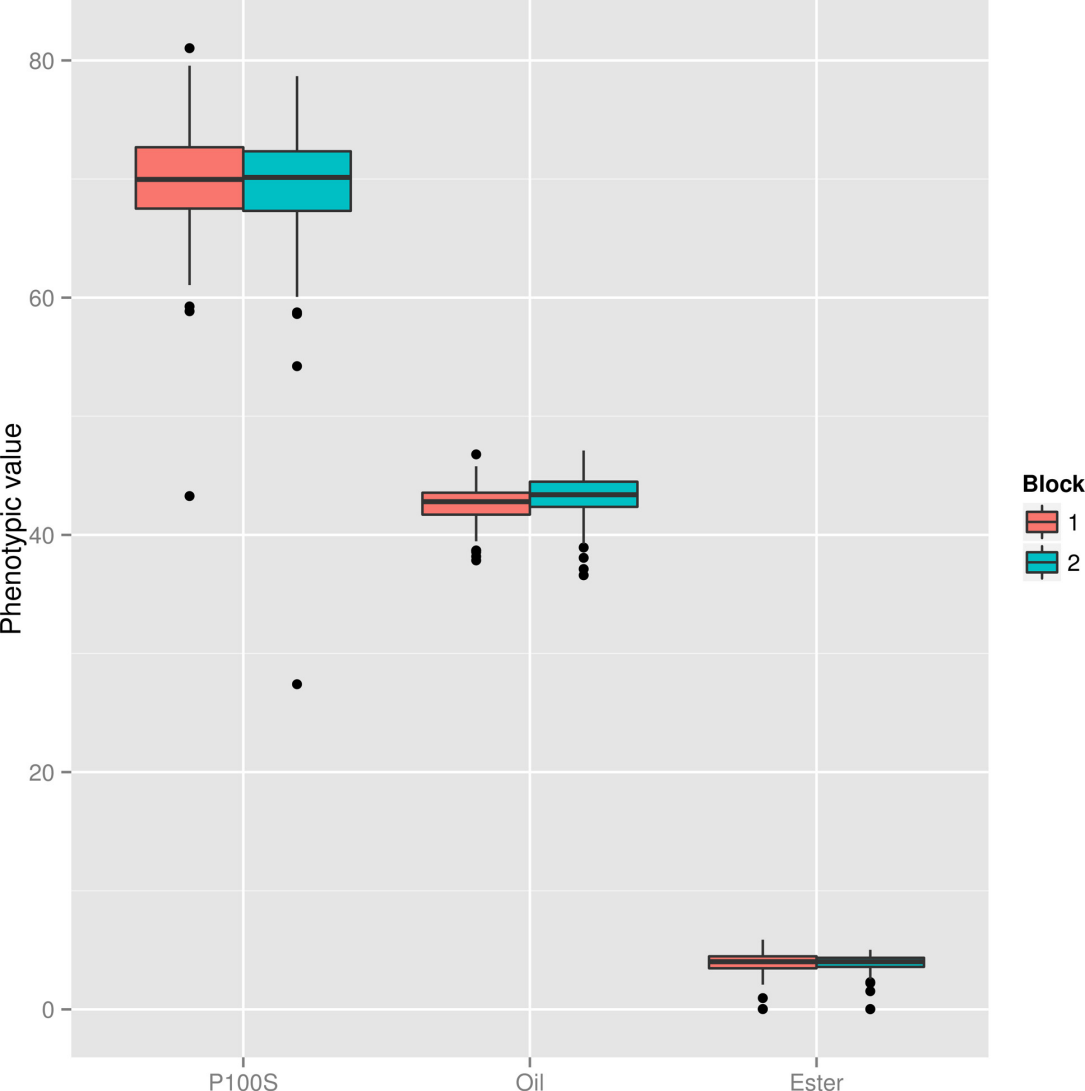
Phenotypic trait evaluation using the Boxplot analysis. Vertical bars are second and third quantiles, and the dots outside the bars are outliers. Each block was evaluated separately, allowing their individual evaluation. **W100S** –weight of 100 seeds; **SOC**–seed oil content; **PEC**–phorbol ester concentration.

We observed that there are no differences between blocks within traits (Fig 1). The highest standard deviation value was observed for **W100S**, followed by **SOC** and **PEC**. We observed a few outlier records for each trait.

### Convergence criterion

Geweke convergence criterion indicates convergence for all dispersion parameters when generating 100,000 MCMC chains, 40,000 samples for burn-in and a sampling interval of 10, totaling 6,000 effective samples used for variance component estimate. Similar posterior mean, median and mode estimates were obtained for variance components, suggesting density with normal shape appearance. Effective sample size (**ESS**) estimated the number of independent samples with information equivalent to that contained within the dependent sampling. Thus, it was observed that the length of the generated chain was adequate since the smallest ESS was 1991.

### Variance components and genetic parameters

Genotypic variance estimated under the Bayesian multi-trait model for **W100S** ($\sigma_{g_{1}}^{2}$) was about 43 times greater than for **SOC** ($\sigma_{g_{2}}^{2}$), or for **PEC** ($\sigma_{g_{3}}^{2}$) (Table 1). The genotypic variances for **SOC** and **PEC** were approximately of the same magnitude. We observed negative covariance between **W100S** and **PEC**, which is a desirable relationship. Covariance between **W100S** and **SOC** was positive, and HPD interval evidences statistical significance of this parameter.

**Table 1:** Variance components and genetic parameters estimated under the Bayesian multi-trait analysis via Gibbs sampling of weight of 100 seeds (W100S), seed oil content (SOC) and phorbol ester concentration (PEC) traits.

Parameter^1^	PM^2^	PMD	PMO	PSD	HPD	Z	ESS
$\sigma_{g_{W100S}}^{2}$	15.522	15.390	14.708	2.061	11.73, 19.65	0.19	5200
$\sigma_{g_{W100S,SOC}}$	0.891	0.884	0.823	0.408	0.10, 1.69	-0.07	4187
$\sigma_{g_{W100S,PEC}}$	-0.319	-0.318	-0.305	0.247	-0.80, 0.17	0.13	5706
$\sigma_{g_{SOC}}^{2}$	0.359	0.337	0.299	0.132	0.15, 0.63	0.15	1991
$\sigma_{g_{SOC,PEC}}$	-0.004	-0.003	0.003	0.067	-0.13, 0.13	0.18	3175
$\sigma_{g_{PEC}}^{2}$	0.373	0.369	0.386	0.059	0.26, 0.49	0.03	6000
$\sigma_{e_{W100S}}^{2}$	7.498	7.446	7.430	0.799	5.95, 9.02	-0.04	6000
$\sigma_{e_{W100S,SOC}}$	0.008	0.004	0.113	0.317	-0.62, 0.63	-0.07	5426
$\sigma_{e_{W100S,PEC}}$	-0.080	-0.079	-0.063	0.116	-0.32, 0.13	-0.21	5203
$\sigma_{e_{SOC}}^{2}$	2.416	2.409	2.452	0.214	1.98, 2.82	-0.11	4190
$\sigma_{e_{SOC,PEC}}$	0.016	0.015	0.020	0.016	-0.11, 0.14	-0.02	4790
$\sigma_{e_{PEC}}^{2}$	0.312	0.310	0.305	0.033	0.25, 0.37	-0.08	5995
$h_{W100S}^{2}$	0.672	0. 674	0.684	0.040	0.586, 0.745	0.131	6000
$h_{SOC}^{2}$	0.129	0.122	0.105	0.045	0.059, 0.232	0.679	2055
$h_{PEC}^{2}$	0.545	0.544	0.551	0.052	0.436, 0.640	-1.573	6000

^1^ Genetic variance of *i*^*th*^ trait ($\sigma_{g_{i}}^{2}$); genetic covariance between traits *i* and *j* ($\sigma_{g_{ij}}$); residual variance of *i*^*th*^ trait ($\sigma_{e_{i}}^{2}$); residual covariance between traits *i* and *j* ($\sigma_{e_{ij}}$); and heritability of *i*^*th*^ trait ($h_{i}^{2}$).

^2^ Posterior mean (PM), posterior median (PMD), posterior mode (PMO), posterior standard deviation (PSD), posterior high density interval (HPD), Z-Geweke (Z) and effective sample size (ESS).

**W100S** presented the highest residual variance (lower certainty), possibly due to a scale effect, followed by **SOC** and **PEC**, respectively (Table 1). Residual covariance estimates among all traits can be considered as not statistically significant when analyzing HPD interval; thus, residual correlations can be considered as absent.

**W100S** and **PEC** presented higher estimated heritabilities than **SOC**. Despite the higher certainty in these estimates being associated to **W100S,** amplitude of HPD intervals was relatively close for all traits (Table 1).

We verified desirable association between **SOC** and **W100S** (positive correlation) for phenotypic and genotypic correlation, being the latter stronger than the former (Table 2). Besides the moderate genotypic association between **SOC** and **W100S**, all the other correlations were of low magnitude for both genotypic and phenotypic correlations. Additionally, desirable negative genotypic correlation was observed between **PEC** and **SOC,** and between **PEC** and **W100S**.

**Table 2:** Heritability (diagonal), genotypic (above) and phenotypic (below) correlation between traits.

Trait	SOC	W100S	PEC
Seed oil content (**SOC**)	0.129	0.544	-0.010
Weight of 100 seeds (**W100S**)	0.113	0.674	-0.130
Phorbol ester concentration (**PEC**)	0.009	-0.101	0.545
Phenotypic variance ${(\sigma }_{p}^{2})$	2.774	23.019	0.685

### Genetic diversity

Genetic diversity analysis aimed to evaluate how the genotypes were distributed between the clusters. In this analysis we used the Ward clustering method based on the Mahalanobis distance, which was calculated using the genotypic values estimated under a Bayesian multi-trait mixed models.

**Fig 2:**
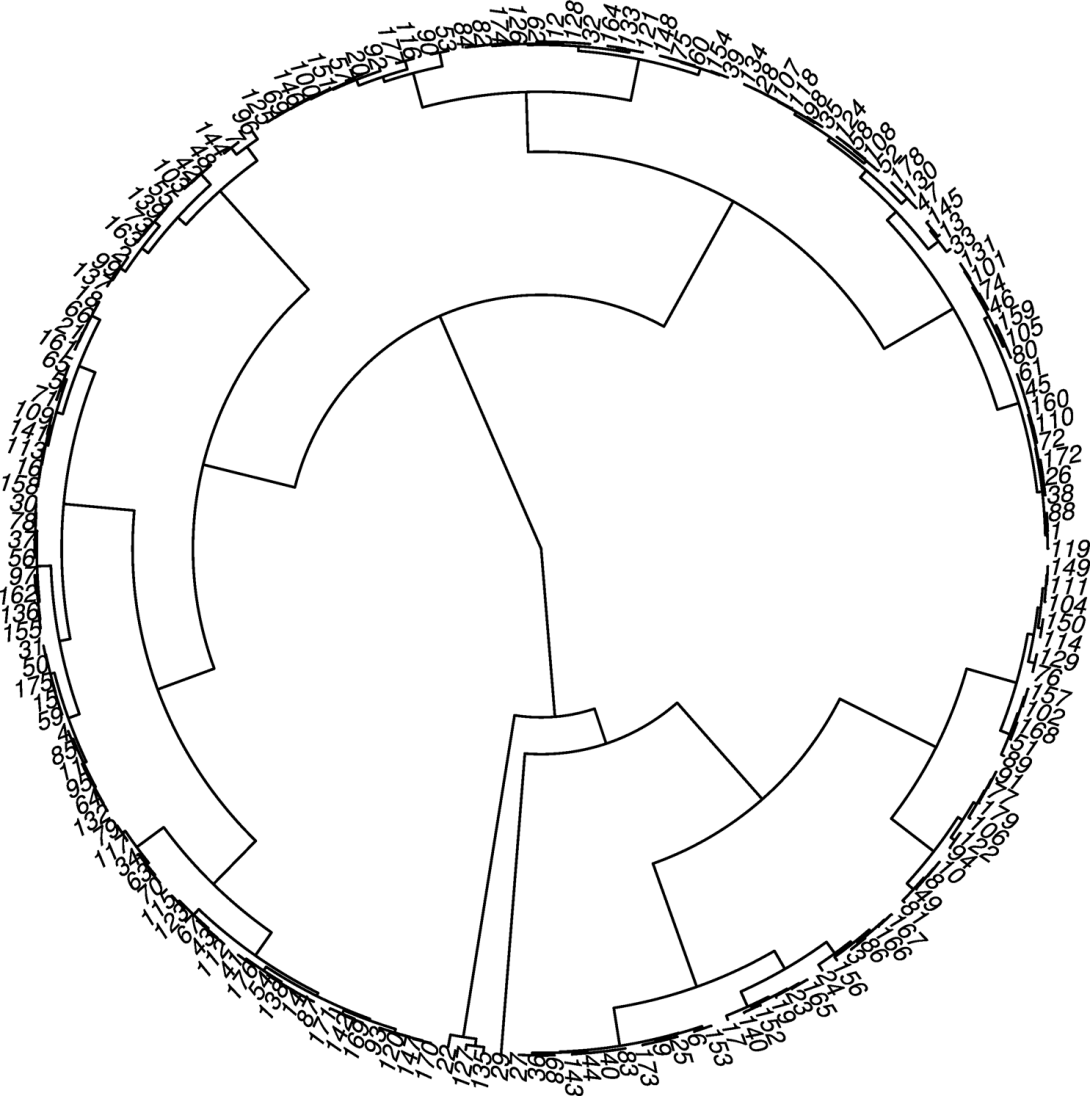
Ward cluster method based on the Mahalanobis distance, calculated using genotypic values estimated by the Bayesian multi-trait analysis.

Nine clusters were suggested according to the Ward method: six big clusters with 35, 31, 28, 23, 24 and 22 half-sib families; one cluster with 13 half-sib families; and two small clusters with 3 and 1 half-sib families (Fig 2).

**Fig 3:**
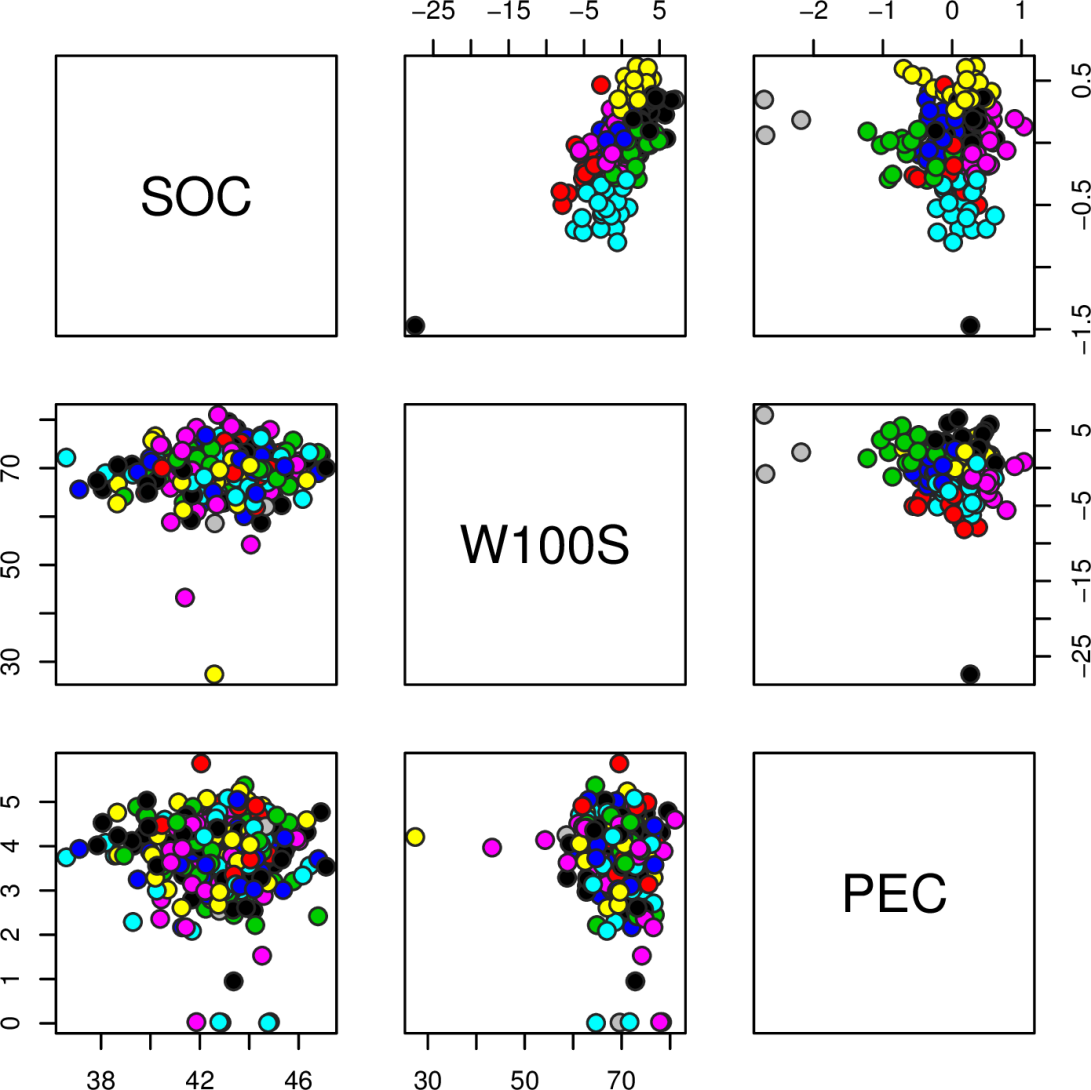
Genotypic values (above diagonal) and phenotypic values (below diagonal) distributions between seed oil content (SOC, g), weight of 100 seed (W100S, %) and phorbol ester concentration (PEC, mg/g).

Phenotypic and genotypic distribution of the nine clusters suggested by the Ward clustering method can be verified in Fig 3. We observed that the use of genotypic values enables a well-defined clustering of genotypes, which is not observed when evaluating the phenotypic values using the clusters suggested by the clustering method. In other words, despite the high heritability, selection of plants based only on phenotypic information could not provide genetic gain for all traits simultaneously.

### Genetic gain and genotype selection based on selection index

After the study of trait’s behavior and after verifying that simultaneous genetic gain will hardly be achieved for all traits, we evaluated several different selection index scenarios (Table 3) aiming to select superior genotypes.

**Table 3:** Scenarios with the respective traits considered by the selection criteria.

Scenario	Traits considered in the selection index^1^
1	SOC
2	SOC + W100S
3	SOC + W100S + PEC

^1^ Weight of 100 seeds (**W100S**, g), Seed oil content (**SOC**, %), and phorbol ester concentration (**PEC**) in seeds (mg/g)

Table 4 shows the results of response to selection per generation (**S**), accuracy of the index, and overall genetic gain among different scenarios and weights. The highest accuracy values were achieved under scenario 3 (all traits were used in selection criteria) regardless of the assessed weights. A different trend was observed for the monetary overall genetic gain per generation (Δ*G*), which was higher when adopting *w3* (quadruple for **SOC**, double for **W100S,** and one for **PEC**) strategies. Additionally, it was possible to obtain simultaneous economically interesting response to selection in scenarios 1 and 2, considering different weighting strategies.

**Table 4:** Response to selection per generation (S), accuracy of the index (R_IH_), and monetary overall genetic gain per generation (Δ*G*) for weight of 100 seeds (W100S, g), seed oil content (SOC, %) and phorbol ester concentration in seeds (PEC, mg/g) using selection index.

			S for each trait		
Scenario^1^	R_IH_	Δ*G*	SOC	W100S	PEC
w1					
1	0.2641	0.8647	0.37806	0.94490	-0.00377
2	0.5866	1.9206	0.42677	5.43193	-0.10412
3	0.7064	2.3128	0.36474	4.45593	0.34990
w2					
1	0.3172	1.4959	0.37806	0.94490	-0.00377
2	0.5631	2.6559	0.45034	5.16525	-0.09691
3	0.6288	2.9657	0.41157	4.58196	0.26170
w3					
1	0.3319	2.9980	0.37806	0.94490	-0.00377
2	0.6058	5.4716	0.44750	5.20812	-0.09802
3	0.6241	5,6372	0.43881	5.03137	0.09332

^1^ In scenario 1 only **SOC** was used as selection criteria. **W100S** was incorporated into the selection index in scenario 2, and **PEC** was added to scenario 3. Economic relative weights (w) were defined as w1 = same, w2 = double for SOC, and w3 = 4(**SOC**), 2(**W100S**) and 1(**PEC**).

## Discussion

### Phenotypic evaluation

Boxplot analysis was carried out to verify how phenotypic data were distributed among blocks. Previous phenotypic analyses are always important for the understanding of each trait, and it helps the researcher in choosing the best way of data evaluation.

Phenotypic variance estimates for seed oil content (**SOC**) and phorbol ester concentration (**PEC**) presented low magnitudes. Different authors have shown the absence of genetic diversity between accessions [13, 29, 30], suggesting that traits’ improvement could be restricted.

The success of the evaluation of a breeding program is related to the accurate prediction of genotypic values, which is closely related to the adoption of proper models. Thus, in this research, we applied a novel statistic approach for variance components estimate under plant breeding schemes. Implementation of the Bayesian multi-trait models is straightforward, and nowadays it has been widely used due to the possibility of considering *a prior* knowledge when modeling. Despite its wide application in animal breeding [31, 32], Bayesian multi-trait analysis has never been reported in plant breeding.

### Variance components and genetic parameters

Genetic and environment parameters estimated under the Bayesian multi-trait approach were similar to **SOC** estimates reported by Peixoto et al. [33], while carrying out analysis of variance (**ANOVA**). Rosado et al. [30] used information of molecular markers and reported low genetic variance estimates among jatropha families. These authors argue that a possible cause would be a common ancestor origin and the selection pressure that this species has suffered in recent years. Indeed, these causes would explain the low genotypic variance observed for **SOC,** and consequently its low heritability. Thereby, selecting superior genotypes based on phenotypic values would not provide an expected overall genetic gain since approximately 83% of the phenotypic variance is not genetic. Thus, it is necessary the adoption of appropriate methodologies to accurately predict genetic effects. Therefore, based on these results and on previous researches, we suggest that the Bayesian multi-trait analysis is more appropriate than ANOVA to perform analysis and select superior genotypes for jatropha breeding, since the Bayesian model can capture minor genetic differences between families, while ANOVA cannot.

The success of a breeding program, which usually aims to improve multiple traits simultaneously, is influenced by the correlation between traits, and mainly by the breeding goal. We observed a non-significant difference for all covariance estimates, except for **SOC** and W100S (Table 1), which suggests that selection based on information of a specific trait will not provide correlated gain to another trait (Table 2). However, it is expected and necessary that multiple traits are improved simultaneously due to the large generation interval of jatropha. Thus, it is necessary the use of statistics techniques that would help breeders to select superior families, and consequently result in reasonable overall gain. Thereby, the use of selection index strategies seems to be a good alternative.

### Genetic diversity

To evaluate the diversity between Jatropha half-sib families, the Ward method was used for clustering, resulting in nine clusters. Singh et al. [34] reported that one of the main problems of jatropha breeding programs is the little genetic variability between genotypes. Moreover, it was reported that the genetic variance is high within families and low between families [29, 35].

We observed low correlation estimates between **W100S** and **SOC** with **PEC**, which is similar to the reports of Peixoto et al. [33]. They estimated correlation based on ANOVA and BLUP results, and observed that there was only significant correlation between **W100S** and **SOC**. Thus, these results mean that when we select for **W100S** we are also selecting for **SOC**, and vice versa. Otherwise, when we select for **W100S** and **SOC,** we are not selecting for **PEC**. Thereby, an option to improve all traits simultaneously is the use of selection index procedures.

### Genetic gain and genotype selection based on selection index

Genotypic values estimated under the Bayesian multi-trait analysis was used to apply selection index procedures, and superior genotypes were selected based on different scenarios.

Despite the low correlation estimates observed between **SOC**, **W100S** and **PEC**, it was possible to achieve overall genetic gain for all traits simultaneously by using selection index. Peixoto et al. [33] used different selection index methods and concluded that a multiplicative index provided genetic gain for all the evaluated traits. Therefore, our work confirms that it is possible to increase **SOC** and reduce **PEC**. This is an important result for jatropha’s breeding, mainly because **PEC** is a limiting factor to the cultivation of this crop.

Simultaneous genetic gain can be achieved by correlated response, which can be maximized with several crossing cycles in order to increase the frequency of favorable alleles for all traits [33]. In this study, we presented a different strategy to achieve simultaneous genetic gain when using the Bayesian multi-traits analysis to estimate genotypic values, and we used them to build a selection index aiming to select superior genotypes.

### Implications and future perspectives

Jatropha is a perennial plant which has been used to produce biofuel, and it has been reported that it is possible to increase its performance by selecting plants based on genetic information. Indeed, the use data of traditional jatropha breeding techniques, novel statistics methods, and molecular markers (i.e. single nucleotide polymorphism, **SNP**) would be the key to improve the accuracy of selection, to reduce the time per cycle, and to decrease the costs per cycle.

In future researches, the use of **SNP** should be exploited aiming to improve prediction accuracy. Recent developments in next-generation sequencing have enabled researchers to quickly and cost-effectively carry out genotyping-by-sequencing of entire breeding populations to discover genetic markers of an entire genome. Therefore, the use of molecular markers for the selection of the best genotypes in breeding populations under field evaluation has recently emerged as the foundation of plant breeding, mainly forest species, since the cycle is too long [36]. Based on theoretical studies and on practical considerations, genomic wide selection (**GWS**) is likely to increase efficiency of breeding programs by shortening the duration of the breeding cycle. Today, progeny testing phase could potentially be omitted, since breeders are able to carry out early selection for yet-to-be observed phenotypes at seedling stage. This early selection would then allow selected individuals being immediately propagated, if micropropagation protocols are available for the immediate establishment of optimized clonal trials with several years of anticipation, compared to the classical breeding scheme [37].

Integration between genomic selection and multi-trait Bayesian approach can increase prediction accuracy. Therefore, selection indices will be more powerful and reliable. Moreover, the identification of chromosome regions that are related to genetic control of multiple traits (pleiotropic genes) would be a useful tool aiming to increase the overall genetic gain during selection. Additionally, the use of molecular data will provide the realized genetic diversity among families since the evaluation will be based on the identical by state (**IBS**) information [38].

## Conclusion

The Bayesian multi-traits analysis integrated with selection indices allowed obtaining selection gain for all traits simultaneously, i.e., it is possible to reduce Jatropha seeds toxicity caused by phorbol ester concentration (**PEC**) and to increase seed oil content (**SOC**).

Based on the estimated genotypic values under the Bayesian multi-trait approach, and on the evaluation of genetic gain when applying the selection index methods, 169 and 170 half-sib families presented high genotypic values for **W100S** and for **SOC,** and low estimates for **PEC**. Thus, these families should be used in future diallel crossings.

## Supporting Information

S1 Table Origin for each accession used in this study. (DOCX)

S2 Table Identification for all jatropha accessions used in this study and the measurements for weight of 100 seeds (W100S, g), seed oil content (SOC, %), and phorbol ester concentration (PEC, mg/g). (DOCX)
