# Correction: Bayesian Multi-Trait Analysis Reveals a Useful Tool to Increase Oil Concentration and to Decrease Toxicity in *Jatropha curcas* L.

**DOI:** 10.1371/journal.pone.0161046

**Published:** 2016-08-08

**Authors:** Vinícius Silva Junqueira, Leonardo de Azevedo Peixoto, Bruno Galvêas Laviola, Leonardo Lopes Bhering, Simone Mendonça, Tania da Silveira Agostini Costa, Rosemar Antoniassi

In Fig 1, the labels for traits use Portuguese abbreviations instead of English. Please see the corrected Fig 1 here.

**Fig 1:**
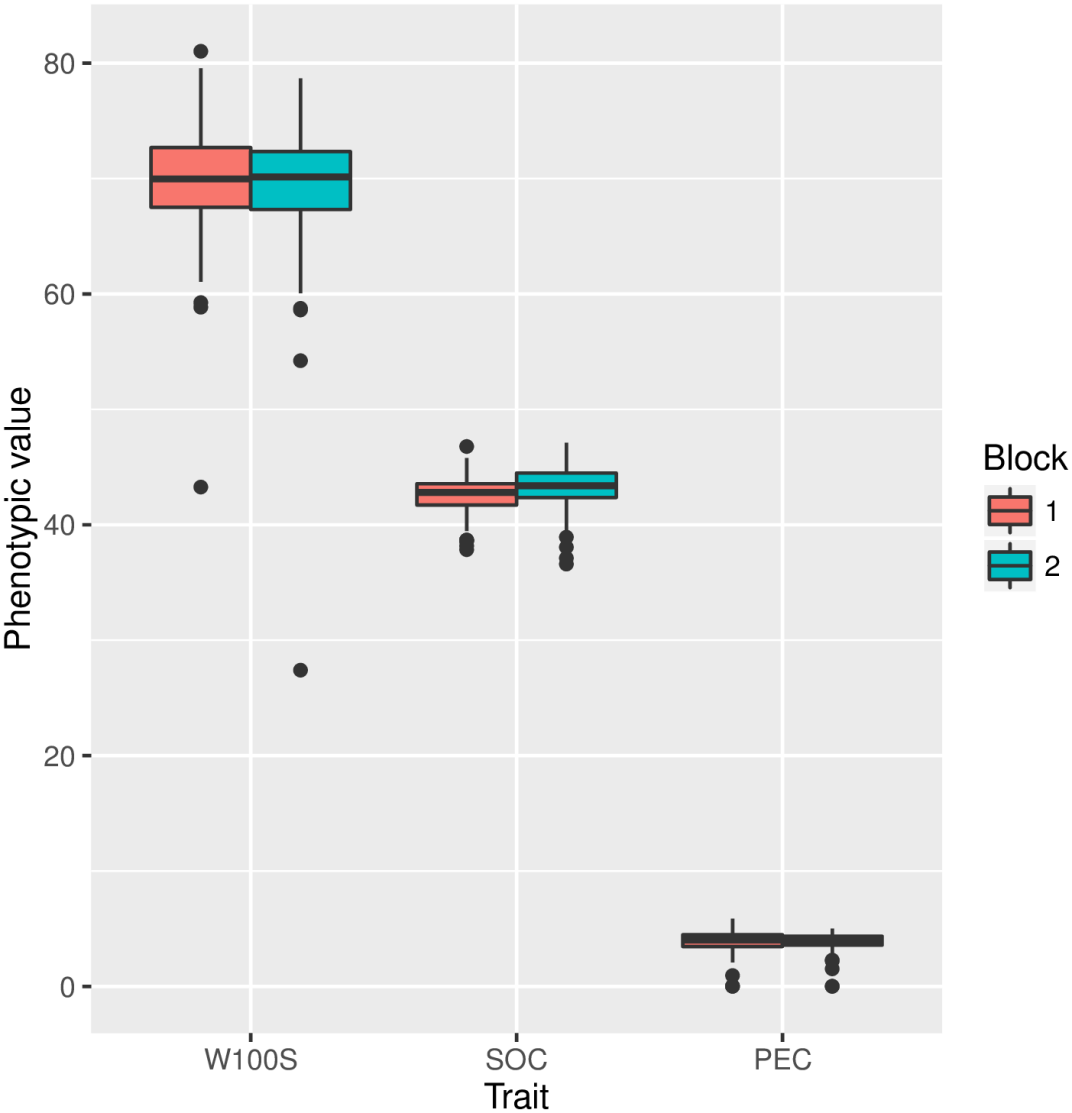
Phenotypic trait evaluation using the Boxplot analysis. Vertical bars are second and third quantiles, and the dots outside the bars are outliers. Each block was evaluated separately, allowing their individual evaluation. **W100S –**weight of 100 seeds; **SOC**–seed oil content; **PEC**–phorbol ester concentration.
