# Supplementary material for: Bayesian Multi-Trait Analysis Reveals a Useful Tool to Increase Oil Concentration and to Decrease Toxicity in Jatropha curcas L
Source: PLoS One. 2016 Jun 9;11(6):e0157038. doi: 10.1371/journal.pone.0157038 (PMC4900661; doi:10.1371/journal.pone.0157038)
Supplement: S2 Table — (DOCX) [file pone.0157038.s002.docx]

**S2 Table**. Identification for all jatropha accessions used in this study and the measurements for weight of 100 seeds (**W100S**, g), seed oil content (**SOC**, %), and phorbol ester concentration (**PEC**, mg/g).

| **Accessions** | **Block** | **W100S** | **SOC** | **PEC** |
| --- | --- | --- | --- | --- |
| 101 | 1 | 79.556 | 43.16015 | 4.79 |
| 101 | 2 | 74.424 | 42.02839 | 4.59 |
| 102 | 1 | 68.606 | 44.0455 | 3.4 |
| 102 | 2 | 74.572 | 40.4573 | 3.35 |
| 103 | 1 | 69.63 | 41.76069 | 3.46 |
| 103 | 2 | 70.844 | 42.46286 | 4.02 |
| 104 | 1 | 69.54 | 42.0578 | 5.87 |
| 104 | 2 | 71.886 | 44.49217 | 4.88 |
| 105 | 1 | 75.726 | 45.78248 | 3.21 |
| 105 | 2 | 71.34 | 43.10049 | 4.34 |
| 106 | 1 | 72.822 | 44.11146 | 4.46 |
| 106 | 2 | 71.524 | 44.75628 | 3.86 |
| 107 | 1 | 71.04 | 41.74785 | 4.08 |
| 107 | 2 | 71.426 | 43.98778 | 4.91 |
| 108 | 1 | 70.812 | 44.47104 | 4.45 |
| 108 | 2 | 74.204 | 44.60294 | 3.1 |
| 110 | 1 | 74.35 | 42.24124 | 2.59 |
| 110 | 2 | 76.014 | 42.47051 | 3.85 |
| 111 | 1 | 76.872 | 43.38031 | 2.45 |
| 111 | 2 | 74.782 | 40.44383 | 2.8 |
| 112 | 1 | 74.12 | 40.4847 | 3.08 |
| 112 | 2 | 74.488 | 44.55583 | 4.04 |
| 113 | 1 | 69.644 | 39.46396 | 4.89 |
| 113 | 2 | 72.718 | 40.53308 | 3.42 |
| 114 | 1 | 70.6 | 43.83412 | 2.81 |
| 114 | 2 | 74.394 | 44.48319 | 3.92 |
| 115 | 1 | 72.77 | 43.88246 | 4.63 |
| 115 | 2 | 71.492 | 42.23713 | 4.59 |
| 116 | 1 | 74.808 | 44.64455 | 4.51 |
| 116 | 2 | 74.036 | 41.81784 | 4 |
| 117 | 1 | 69.84 | 43.82657 | 3.09 |
| 117 | 2 | 71.426 | 46.01826 | 4.12 |
| 118 | 1 | 77.278 | 43.55834 | 4.23 |
| 118 | 2 | 70.714 | 42.41222 | 3.31 |
| 120 | 1 | 78.156 | 43.49103 | 4.31 |
| 120 | 2 | 70.858 | 45.58696 | 4.47 |
| 121 | 1 | 77.034 | 43.49275 | 3.99 |
| 121 | 2 | 72.326 | 44.3757 | 4.26 |
| 122 | 1 | 81.032 | 42.73889 | 4.6 |
| 122 | 2 | 74.896 | 43.49601 | 3.51 |
| 123 | 1 | 73.264 | 43.94693 | 5.03 |
| 123 | 2 | 77.184 | 43.35001 | 4.06 |
| 124 | 1 | 70.688 | 43.14414 | 4.48 |
| 124 | 2 | 72.14 | 43.97102 | 4.18 |
| 125 | 1 | 71.032 | 40.75277 | 4.06 |
| 125 | 2 | 72.04 | 43.76135 | 4.12 |
| 126 | 1 | 69.588 | 41.98743 | 3.77 |
| 126 | 2 | 73.54 | 44.13623 | 3.65 |
| 127 | 1 | 68.92 | 44.47561 | 3.31 |
| 127 | 2 | 66.5528 | 41.36182 | 3.93 |
| 128 | 1 | 69.28 | 38.55774 | 3.79 |
| 128 | 2 | 69.58 | 39.30929 | 4.01 |
| 129 | 1 | 73.804 | 43.55042 | 3.87 |
| 129 | 2 | 70.798 | 40.70155 | 3.02 |
| 130 | 1 | 75.184 | 43.22274 | 4.91 |
| 130 | 2 | 76.338 | 43.89691 | 4.27 |
| 131 | 1 | 67.426 | 41.91835 | 4.61 |
| 131 | 2 | 65.654 | 38.06479 | 4.53 |
| 132 | 1 | 73.888 | 43.12008 | 3.62 |
| 132 | 2 | 71.886 | 41.13797 | 4.36 |
| 133 | 1 | 73.62 | 43.74163 | 4.57 |
| 133 | 2 | 73.49 | 45.41874 | 4.36 |
| 134 | 1 | 64.208 | 43.917 | 4.49 |
| 134 | 2 | 62.852 | 41.78442 | 3.11 |
| 136 | 1 | 68.008 | 44.95713 | 4.48 |
| 136 | 2 | 68.88 | 42.94654 | 4.23 |
| 137 | 1 | 67.5 | 42.45753 | 2.8 |
| 137 | 2 | 67.424 | 43.68936 | 4 |
| 138 | 1 | 66.81 | 43.60032 | 4.69 |
| 138 | 2 | 61.96 | 44.19369 | 4.73 |
| 139 | 1 | 72.48 | 43.05474 | 3.72 |
| 139 | 2 | 0 | 44.33528 | 4.74 |
| 140 | 1 | 73.716 | 42.33687 | 4.23 |
| 140 | 2 | 73.048 | 44.69692 | 4.75 |
| 141 | 1 | 72.74 | 42.46459 | 4.74 |
| 141 | 2 | 74.832 | 44.36392 | 4 |
| 142 | 1 | 69.634 | 42.11365 | 3.46 |
| 142 | 2 | 60.07 | 43.79695 | 3.92 |
| 143 | 1 | 73.366 | 44.16568 | 4.36 |
| 143 | 2 | 71.562 | 44.69289 | 3.12 |
| 145 | 1 | 71.716 | 40.26085 | 3 |
| 145 | 2 | 70.488 | 42.9791 | 4.57 |
| 147 | 1 | 74.482 | 41.47054 | 3.51 |
| 147 | 2 | 70.12 | 44.5915 | 2.88 |
| 148 | 1 | 70.15 | 41.31361 | 3.78 |
| 148 | 2 | 67.404 | 42.0983 | 3.23 |
| 149 | 1 | 76.62 | 40.20144 | 3.29 |
| 149 | 2 | 71.184 | 44.18254 | 2.55 |
| 150 | 1 | 67.966 | 40.58076 | 4.6 |
| 150 | 2 | 67.798 | 44.00461 | 4.13 |
| 151 | 1 | 72.026 | 43.58883 | 4.78 |
| 151 | 2 | 70.592 | 43.43158 | 4.01 |
| 152 | 1 | 72.612 | 41.13043 | 4.99 |
| 152 | 2 | 67.172 | 43.03237 | 3.9 |
| 153 | 1 | 72.098 | 39.98023 | 3.88 |
| 153 | 2 | 73.482 | 43.53499 | 4.84 |
| 154 | 1 | 69.754 | 42.52464 | 4.68 |
| 154 | 2 | 72.736 | 46.90334 | 4.77 |
| 155 | 1 | 70.744 | 43.55566 | 4.72 |
| 155 | 2 | 72.454 | 44.797 | 4.1 |
| 156 | 1 | 69.956 | 43.10272 | 3.2 |
| 156 | 2 | 68.284 | 43.36284 | 4 |
| 157 | 1 | 69.654 | 42.88118 | 4.98 |
| 157 | 2 | 74.432 | 43.67274 | 3 |
| 158 | 1 | 68.53 | 42.98243 | 3.29 |
| 158 | 2 | 67.246 | 42.7178 | 3.1 |
| 159 | 1 | 72.01 | 41.32228 | 3.35 |
| 159 | 2 | 72.294 | 41.67088 | 3.79 |
| 160 | 1 | 68.574 | 43.78427 | 3.67 |
| 160 | 2 | 65.488 | 43.31238 | 3.69 |
| 161 | 1 | 73.274 | 43.37827 | 3.7 |
| 161 | 2 | 71.118 | 45.02282 | 4.64 |
| 163 | 1 | 72.092 | 41.28264 | 2.17 |
| 163 | 2 | 76.616 | 42.82914 | 2.71 |
| 164 | 1 | 75.718 | 40.07683 | 3.8 |
| 164 | 2 | 78.674 | 43.28159 | 3.89 |
| 165 | 1 | 66.746 | 38.6847 | 3.8 |
| 165 | 2 | 71.844 | 42.62586 | 4.38 |
| 166 | 1 | 74.174 | 43.24242 | 4.27 |
| 166 | 2 | 63.678 | 44.12885 | 3.67 |
| 167 | 1 | 65.82 | 42.50372 | 4.28 |
| 167 | 2 | 72.198 | 36.59709 | 3.75 |
| 168 | 1 | 73.504 | 43.0381 | 4.6 |
| 168 | 2 | 68.894 | 42.21955 | 3.38 |
| 169 | 1 | 78.376 | 41.8652 | 0.03 |
| 169 | 2 | 77.932 | 44.84547 | 0.03 |
| 170 | 1 | 69.592 | 42.88185 | 0.02 |
| 170 | 2 | 74.19738 | 44.52424 | 1.53 |
| 172 | 1 | 68.97 | 38.18199 | 4.08 |
| 172 | 2 | 65.638 | 43.31954 | 3 |
| 174 | 1 | 63.774 | 45.01979 | 3.59 |
| 174 | 2 | 65.5042 | 42.31056 | 2.89 |
| 175 | 1 | 72.546 | 43.24709 | 3.95 |
| 175 | 2 | 70.778 | 42.81949 | 3.98 |
| 176 | 1 | 71.02 | 41.78992 | 4.11 |
| 176 | 2 | 71.498 | 43.36746 | 4.22 |
| 177 | 1 | 69.328 | 42.37792 | 4.37 |
| 177 | 2 | 67.998 | 43.8281 | 4.18 |
| 178 | 1 | 73.156 | 45.203 | 3.84 |
| 178 | 2 | 67.204 | 44.99731 | 3.2 |
| 179 | 1 | 66.576 | 43.28609 | 4.66 |
| 179 | 2 | 67.438 | 43.2365 | 3.97 |
| 180 | 1 | 64.578 | 43.81587 | 5.37 |
| 180 | 2 | 75.32576 | 43.67531 | 4.99 |
| 181 | 1 | 64.84 | 42.47659 | 4.27 |
| 181 | 2 | 65.56 | 41.99838 | 3.7 |
| 182 | 1 | 69.976 | 42.2887 | 5.07 |
| 182 | 2 | 71.09 | 43.38508 | 4.5 |
| 183 | 1 | 71.65939 | 42.79172 | 0.03 |
| 183 | 2 | 64.73 | 44.76492 | 0.01 |
| 185 | 1 | 65.976 | 40.80996 | 3.91 |
| 185 | 2 | 66.676 | 42.94191 | 3.42 |
| 186 | 1 | 70.648 | 43.13699 | 2.91 |
| 186 | 2 | 67.64 | 44.79462 | 3.88 |
| 187 | 1 | 73.8946 | 44.73826 | 4.41 |
| 187 | 2 | 73.456 | 45.51277 | 3.98 |
| 188 | 1 | 73.144 | 42.80289 | 4.46 |
| 188 | 2 | 76.75379 | 44.48034 | 4.46 |
| 189 | 1 | 68.566 | 39.75373 | 4.25 |
| 189 | 2 | 71.67 | 42.29541 | 4 |
| 190 | 1 | 63.946 | 43.02189 | 4.15 |
| 190 | 2 | 67.9126 | 45.37675 | 3.01 |
| 191 | 1 | 67.246 | 43.30031 | 4.96 |
| 191 | 2 | 70.472 | 44.6207 | 4.49 |
| 192 | 1 | 68.342 | 41.35754 | 3.53 |
| 192 | 2 | 72.47863 | 43.36783 | 4.01 |
| 193 | 1 | 66.868 | 42.62759 | 3.79 |
| 193 | 2 | 67.842 | 44.5269 | 3.61 |
| 194 | 1 | 71.168 | 43.63251 | 5.24 |
| 194 | 2 | 72.008 | 44.94823 | 3.99 |
| 195 | 1 | 71.488 | 41.81745 | 2.91 |
| 195 | 2 | 71.742 | 42.80267 | 3.09 |
| 196 | 1 | 73.175 | 44.37914 | 3.99 |
| 196 | 2 | 69.552 | 45.99831 | 4.41 |
| 198 | 1 | 71.742 | 44.08204 | 3.58 |
| 198 | 2 | 70.684 | 40.30356 | 4.24 |
| 199 | 1 | 69.452 | 42.7486 | 4.25 |
| 199 | 2 | 66.256 | 41.92393 | 4.28 |
| 200 | 1 | 62.812 | 42.09062 | 4.34 |
| 200 | 2 | 70.736 | 39.25563 | 4.12 |
| 201 | 1 | 64.906 | 39.79259 | 4.69 |
| 201 | 2 | 66.3246 | 45.61402 | 4.53 |
| 202 | 1 | 72.2246 | 44.47573 | 3.43 |
| 202 | 2 | 71.016 | 44.46815 | 4.32 |
| 203 | 1 | 67.074 | 41.72391 | 4.89 |
| 203 | 2 | 68.76703 | 44.83249 | 4.42 |
| 204 | 1 | 61.4 | 41.47381 | 4.71 |
| 204 | 2 | 72.955 | 43.57544 | 4.46 |
| 205 | 1 | 70.598 | 43.49866 | 4.14 |
| 205 | 2 | 70.106 | 42.00844 | 4.44 |
| 206 | 1 | 70.962 | 42.26226 | 4.04 |
| 206 | 2 | 73.174 | 42.70531 | 4.76 |
| 207 | 1 | 67.702 | 44.03043 | 3.75 |
| 207 | 2 | 65.62 | 37.1209 | 3.94 |
| 208 | 1 | 62.908 | 42.12496 | 3.3 |
| 208 | 2 | 64.134 | 38.93382 | 3.79 |
| 209 | 1 | 68.318 | 43.67977 | 3.72 |
| 209 | 2 | 68.404 | 39.86283 | 4.68 |
| 210 | 1 | 70.804 | 42.49854 | 4.59 |
| 210 | 2 | 73.606 | 44.67663 | 4.21 |
| 211 | 1 | 72.854 | 42.79744 | 2.54 |
| 211 | 2 | 68.112 | 41.97115 | 3.35 |
| 212 | 1 | 65.2 | 44.05251 | 4.69 |
| 212 | 2 | 72.156 | 43.84501 | 4.34 |
| 214 | 1 | 73.76 | 43.26745 | 3.31 |
| 214 | 2 | 73.996 | 41.58913 | 4.24 |
| 215 | 1 | 69.96 | 43.86758 | 3.71 |
| 215 | 2 | 68.748 | 44.68335 | 4.18 |
| 216 | 1 | 71.882 | 43.36464 | 3.12 |
| 216 | 2 | 65.99 | 43.67532 | 3.97 |
| 217 | 1 | 70.484 | 43.06006 | 4.83 |
| 217 | 2 | 74.656 | 43.34997 | 2.57 |
| 218 | 1 | 68.968 | 44.53235 | 4.91 |
| 218 | 2 | 66.238 | 42.93509 | 4.35 |
| 219 | 1 | 72.5 | 43.94441 | 4.54 |
| 219 | 2 | 73.086 | 43.02565 | 4.32 |
| 220 | 1 | 67.75 | 43.05181 | 4.08 |
| 220 | 2 | 65.198 | 44.86321 | 3.76 |
| 221 | 1 | 72.68 | 42.93978 | 3.08 |
| 221 | 2 | 67.032 | 43.56153 | 4.16 |
| 222 | 1 | 72.682 | 42.25537 | 3.64 |
| 222 | 2 | 71.748 | 44.76344 | 3.17 |
| 223 | 1 | 73.17 | 46.78892 | 2.42 |
| 223 | 2 | 71.648 | 44.14526 | 3.36 |
| 224 | 1 | 71.848 | 44.91052 | 4.47 |
| 224 | 2 | 71.65067 | 46.32965 | 4.32 |
| 225 | 1 | 75.598 | 43.00687 | 3.14 |
| 225 | 2 | 72.352 | 45.29394 | 3.71 |
| 226 | 1 | 70.936 | 43.53037 | 4.43 |
| 226 | 2 | 68.676 | 39.29402 | 2.29 |
| 227 | 1 | 67.346 | 42.23028 | 3.73 |
| 227 | 2 | 68.91 | 43.27729 | 4.86 |
| 228 | 1 | 72.826 | 42.61372 | 4.03 |
| 228 | 2 | 73.156 | 43.57402 | 4.89 |
| 229 | 1 | 72.338 | 43.54827 | 4.05 |
| 229 | 2 | 70.784 | 45.91497 | 4.17 |
| 230 | 1 | 74.482 | 44.20188 | 4.13 |
| 230 | 2 | 67.386 | 42.79011 | 4.08 |
| 231 | 1 | 74.808 | 40.39957 | 2.36 |
| 231 | 2 | 70.142 | 41.68369 | 2.8 |
| 232 | 1 | 71.232 | 41.78596 | 4.16 |
| 232 | 2 | 64.796 | 42.71389 | 4.2 |
| 233 | 1 | 69.204 | 39.49011 | 3.25 |
| 233 | 2 | 72.51 | 44.50573 | 4.24 |
| 234 | 1 | 70.764 | 41.61353 | 3.84 |
| 234 | 2 | 67.744 | 42.70639 | 4.45 |
| 235 | 1 | 68.64 | 40.49778 | 3.56 |
| 235 | 2 | 65.734 | 42.53249 | 3.83 |
| 236 | 1 | 72.722 | 42.93214 | 3.74 |
| 236 | 2 | 68.854 | 43.58728 | 5.02 |
| 237 | 1 | 72.84 | 43.37148 | 0.95 |
| 237 | 2 | 69.354 | 43.09045 | 3.32 |
| 238 | 1 | 70.584 | 38.68372 | 4.23 |
| 238 | 2 | 69.078 | 41.37543 | 3.97 |
| 239 | 1 | 72.628 | 43.12859 | 5.08 |
| 239 | 2 | 70.888 | 43.48458 | 4.3 |
| 240 | 1 | 71.344 | 40.0308 | 4.44 |
| 240 | 2 | 68.06678 | 43.22434 | 4.28 |
| 241 | 1 | 67.53 | 40.27225 | 3.56 |
| 241 | 2 | 64.41 | 42.15045 | 4.02 |
| 243 | 1 | 68.498 | 42.53648 | 3.65 |
| 243 | 2 | 66.616 | 43.17494 | 3.48 |
| 244 | 1 | 71.01 | 41.14518 | 3.65 |
| 244 | 2 | 72.516 | 41.72844 | 3.85 |
| 247 | 1 | 71.262 | 42.65064 | 4.07 |
| 247 | 2 | 68.326 | 42.99882 | 3 |
| 250 | 1 | 73.292 | 43.87515 | 2.61 |
| 250 | 2 | 73.26 | 46.44959 | 3.56 |
| 251 | 1 | 68.948 | 42.281 | 4.53 |
| 251 | 2 | 64.852 | 42.75971 | 4.77 |
| 252 | 1 | 68.588 | 41.08681 | 4.4 |
| 252 | 2 | 67.088 | 42.67448 | 4.2 |
| 253 | 1 | 72.156 | 43.02594 | 3 |
| 253 | 2 | 70.058 | 45.02652 | 3.89 |
| 254 | 1 | 69.948 | 44.84129 | 3.8 |
| 254 | 2 | 70.126 | 41.19703 | 3.58 |
| 255 | 1 | 66.646 | 39.85562 | 5.03 |
| 255 | 2 | 70.026 | 40.47 | 4.48 |
| 256 | 1 | 71.078 | 42.62867 | 4.17 |
| 256 | 2 | 72.688 | 43.60842 | 4.35 |
| 257 | 1 | 67.068 | 44.75038 | 4.77 |
| 257 | 2 | 68.222 | 44.3991 | 4.7 |
| 258 | 1 | 67.934 | 43.47217 | 3.5 |
| 258 | 2 | 69.546 | 45.43608 | 3.87 |
| 260 | 1 | 69.118 | 44.63904 | 4.15 |
| 260 | 2 | 70.004 | 41.42527 | 4.68 |
| 261 | 1 | 62.358 | 45.3159 | 3.7 |
| 261 | 2 | 68.992 | 46.8001 | 3.71 |
| 262 | 1 | 67.412 | 37.85059 | 4.02 |
| 262 | 2 | 63.632 | 46.16963 | 3.34 |
| 263 | 1 | 67.6 | 44.02435 | 4.35 |
| 263 | 2 | 69.06 | 41.41359 | 4.45 |
| 264 | 1 | 72.848 | 42.76699 | 3.55 |
| 264 | 2 | 67.49 | 46.32941 | 4.59 |
| 265 | 1 | 73.5 | 43.29568 | 3.78 |
| 265 | 2 | 71.854 | 43.30915 | 4.15 |
| 266 | 1 | 64.692 | 44.29709 | 4.46 |
| 266 | 2 | 58.76 | 44.48363 | 3.29 |
| 267 | 1 | 62.716 | 38.6586 | 4.76 |
| 267 | 2 | 63.906 | 43.42796 | 3.54 |
| 268 | 1 | 62.856 | 42.646 | 4.63 |
| 268 | 2 | 64.39156 | 41.73959 | 4.05 |
| 269 | 1 | 62.768 | 42.84182 | 2.97 |
| 269 | 2 | 63.802 | 42.79515 | 3.16 |
| 270 | 1 | 61.062 | 41.88116 | 4.51 |
| 270 | 2 | 58.62 | 42.60975 | 4.25 |
| 271 | 1 | 66.964 | 41.68881 | 3.14 |
| 271 | 2 | 66.152 | 43.97419 | 3.57 |
| 272 | 1 | 59.26 | 41.64002 | 4.06 |
| 272 | 2 | 65.044 | 39.91181 | 4.44 |
| 274 | 1 | 64.54 | 41.73868 | 4.02 |
| 274 | 2 | 56.1875 | 44.06766 | 4.14 |
| 275 | 1 | 62.906 | 42.98647 | 3.26 |
| 275 | 2 | 62.052 | 44.66605 | 4.45 |
| 276 | 1 | 63.208 | 43.51693 | 5.05 |
| 276 | 2 | 61.92 | 44.28065 | 4.91 |
| 277 | 1 | 62.506 | 43.53373 | 3.66 |
| 277 | 2 | 67.638 | 42.28543 | 4.14 |
| 278 | 1 | 62.912 | 41.34705 | 4.51 |
| 278 | 2 | 65.102 | 42.54208 | 4.02 |
| 279 | 1 | 61.36 | 41.3335 | 4.06 |
| 279 | 2 | 64.908 | 44.24696 | 2.22 |
| 280 | 1 | 68.972 | 43.37333 | 3.36 |
| 280 | 2 | 69.56 | 42.07426 | 3.94 |
| 281 | 1 | 58.53667 | 41.40155 | 3.97 |
| 281 | 2 | 0 | 42.58896 | 4.21 |
| 282 | 1 | 58.855 | 40.83049 | 3.63 |
| 282 | 2 | 62.734 | 44.18822 | 4.42 |
| 283 | 1 | 64.1166 | 43.45725 | 3.14 |
| 283 | 2 | 62.486 | 42.68562 | 4.41 |
| 289 | 1 | 69.878 | 44.53973 | 3.88 |
| 289 | 2 | 67.806 | 45.12438 | 4.69 |
| 290 | 1 | 69.026 | 42.21935 | 2.99 |
| 290 | 2 | 71.86 | 43.63667 | 3.1 |
| 297 | 1 | 72.05 | 41.89694 | 4.09 |
| 297 | 2 | 76.17 | 44.47752 | 4.05 |
| 298 | 1 | 69.26689 | 44.19297 | 3.03 |
| 298 | 2 | 70.086 | 47.11428 | 3.54 |
| 299 | 1 | 64.334 | 41.68995 | 4.36 |
| 299 | 2 | 70.38 | 45.43767 | 4.19 |
| 300 | 1 | 64.782 | 44.28549 | 3.73 |
| 300 | 2 | 70.405 | 44.05359 | 3.7 |
| 302 | 1 | 69.572 | 41.69423 | 4.49 |
| 302 | 2 | 74.062 | 42.48377 | 3.9 |
| 303 | 1 | 70.724 | 42.43616 | 3.61 |
| 303 | 2 | 69.762 | 44.39297 | 4.12 |
| 304 | 1 | 66.98105 | 41.68608 | 2.09 |
| 304 | 2 | 70.58 | 44.03862 | 4.04 |
| 309 | 1 | 67.08644 | 41.26148 | 2.61 |
| 309 | 2 | 69.328 | 42.75436 | 2.68 |
| 310 | 1 | 69.6025 | 41.32928 | 4.27 |
| 310 | 2 | 71.832 | 41.07808 | 4.54 |
| 312 | 1 | 76.58111 | 41.4395 | 2.17 |
| 312 | 2 | 76.788 | 42.25112 | 3.58 |
| 313 | 1 | 69.66 | 42.80532 | 2.97 |
| 313 | 2 | 73.61 | 41.29243 | 3.95 |
| 315 | 1 | 0 | 42.73331 | 4.4 |
| 315 | 2 | 68.192 | 42.17844 | 4.22 |
